# Large-scale production of a thermostable *Rhodothermus marinus* cellulase by heterologous secretion from *Streptomyces lividans*

**DOI:** 10.1186/s12934-017-0847-x

**Published:** 2017-12-23

**Authors:** Mohamed Belal Hamed, Spyridoula Karamanou, Solveig Ólafsdottir, Joana Sofia Martins Basílio, Kenneth Simoens, Kostantinos C. Tsolis, Lieve Van Mellaert, Eik Elísabet Guðmundsdóttir, Gudmundur Oli Hreggvidsson, Jozef Anné, Kristel Bernaerts, Olafur H. Fridjonsson, Anastassios Economou

**Affiliations:** 1 Department of Microbiology and Immunology, Rega Institute, KU Leuven, Herestraat 49, 3000 Louvain, Belgium; 2 Matís, Vínlandsleid 12, 113 Reykjavík, Iceland; 3 Bio- & Chemical Systems Technology, Reactor Engineering and Safety Section, Department of Chemical Engineering, KU Leuven, Celestijnenlaan 200F, 3001 Louvain, Belgium; 4 Department of Molecular Biology, National Research Centre, Dokki, Giza Egypt

**Keywords:** Cellulase, Protein translocase, Signal peptide, Secretion, *Streptomyces lividans*, Protein secretion biotechnology

## Abstract

**Background:**

The gene encoding a thermostable cellulase of family 12 was previously isolated from a *Rhodothermus marinus* through functional screening. CelA is a protein of 260 aminoacyl residues with a 28-residue amino-terminal signal peptide. Mature CelA was poorly synthesized in some *Escherichia coli* strains and not at all in others. Here we present an alternative approach for its heterologous production as a secreted polypeptide in *Streptomyces*.

**Results:**

CelA was successfully over-expressed as a secreted polypeptide in *Streptomyces lividans* TK24. To this end, CelA was fused C-terminally to the secretory signal peptide of the subtilisin inhibitor protein (Sianidis et al. in J Biotechnol. 121: 498–507, 2006) from *Streptomyces venezuelae* and a new cloning strategy developed. Optimal growth media and conditions that stall biomass production promote excessive CelA secretion. Under optimal growth conditions in nutrient broth medium, significant amounts of mature CelA (50–90 mg/L or 100–120 mg/g of dry cell weight) are secreted in the spent growth media after 7 days. A protocol to rapidly purify CelA to homogeneity from culture supernatants was developed and specific anti-sera raised against it. Biophysical, biochemical and immmuno-detection analyses indicate that the enzyme is intact, stable and fully functional. CelA is the most thermostable heterologous polypeptide shown to be secreted from *S. lividans*.

**Conclusion:**

This study further validates and extends the use of the *S. lividans* platform for production of heterologous enzymes of industrial importance and extends it to active thermostable enzymes. This study contributes to developing a platform for poly-omics analysis of protein secretion in *S. lividans*.

**Electronic supplementary material:**

The online version of this article (10.1186/s12934-017-0847-x) contains supplementary material, which is available to authorized users.

## Background

*Streptomyces lividans* has been used for the heterologous secretion of several polypeptides of bacterial and eukaryotic origin (for examples see [1–5]). Commonly, heterologous genes are fused to signal peptide sequences from highly expressed/secreted endogenous *Streptomyces* proteins [6–8]. The resulting proteins are thus targeted to the *S. lividans* Sec pathway and very efficiently secreted directly into the growth medium. The absence of lipopolysaccharides, the advanced genetic manipulation tools [9], the established bioprocessing regimes, the low protease activity and the avoidance of inclusion body formation, render *S. lividans* secretion an attractive biotechnology platform. In many instances, it can provide alternative solutions when established workhorses, like *Escherichia coli*, fail or are more costly in bio-processing terms [8]. Moreover, *S. lividans* is being developed as a powerful experimental system on which poly-omics tools can be applied to gain understanding on the molecular underpinnings of protein secretion regulation.

Previously, we demonstrated that *S. lividans* can efficiently secrete active trimeric murine tumor necrosis factor alpha (mTNFα) [5, 10], a *Jonesia* sp. xyloglucanase of 100 kDa [1] and other polypeptides such as phospholipase D [3], transglutaminase (TGase), β-1,4-endoglucanase and β-glucosidase [11] into the growth medium. One successful approach has been the use of the transcription elements and the signal peptide of the *Streptomyces venezuelae* CBS762.70 subtilisin inhibitor gene [12] (hereafter: *vsi*).

We have now extended the use of the *S. lividans* secretion system for the production of thermostable enzymes of industrial interest, using as an example a cellulase of glycosyl hydrolase family 12 (CelA) from *Rhodothermus marinus* [13]. The enzyme was previously shown to have activity on carboxymethyl cellulose and lichenan, but not on birch xylan or laminarin with a pH optimum of 6–7 and its highest measured initial activity was at 100 °C. Its structure has been solved by X-ray crystallography and revealed a beta-jelly roll fold, with an identical topology to those of mesophilic members of the family like that of the endogenous CelB2 of *S. lividans* [14], with an elongated groove that binds the substrates [15]. The mature region of CelA (residues 29–233) fused behind the SP^vsi^ is efficiently secreted from *S. lividans* as a discrete polypeptide of ~ 26 kDa (expected size 26.05 kDa) in amounts exceeding 40 mg/L when grown in nutrient broth or double strength NB media for 48 h. Yields of 50–90 mg/L or 100–120 mg/g of dry cell weight can be produced after 7 days. Various media tested show significant differences in amounts of secreted protein. One characteristic observation is that the best performing media for secretion give the lowest amounts of biomass and unabated secretion at late growth phases. Isolation of the enzyme revealed it to be catalytically functional and stable. These data extend the use of the *S. lividans* secretion biotechnology platform to the production of thermostable enzymes of industrial importance and set the stage for a rational understanding of the molecular basis of protein secretion regulation using poly-omics approaches.

## Methods

### Bacterial strains and recombinant DNA experiments

Growth and manipulation of *E. coli* and *Streptomyces* strains were as described [9, 16].

### Cloning of _*Rm*_*celA* in *S. lividans* TK24

The native _*Rm*_ *celA* gene was amplified from *R. marinus* and ligated as a blunt end—*Pst*I fragment into pBSDK0.6Sma [10] following *Dra*II digest, Klenow modification, and a subsequent *Pst*I digest. pBSDK0.6Sma is used to construct *vsi*-*celA*. CelA (also called Cel12A, a superfamily 12 of hydrolases) devoid of its predicted signal peptide (aa residues 29–263) was fused C-terminally to the signal peptide of Vsi (SP^Vsi^), the subtilisin inhibitor of *S. venezuelae CBS762.70* [12]. Two additional amino acids of the mature Vsi domain were maintained in the fusion protein SP^Vsi^-EA-CelA_29–263_ (Additional file 1: Figure S1). Its expression was placed under the control of the strong constitutive *vsi* gene promoter. To this end, the region of *celA* that encodes residues 29–263 was amplified by PCR using the primers *celA*-*dsp*-*f* (5′-AAGGAACCGGAGCCTGAG) and *celA*-*pst*-*mfe*-*r* (5′-AAAACTGCAGACAATTGCTACTGCACCGTTACGGAAAAATC) and genomic DNA isolated from *R. marinus* DSM4253 as a template. No restriction site was included in the forward primer, while a *Pst*I and a *Mfe*I site were introduced in the 5′ end of the reverse primer.

The resulting plasmid containing *celA* downstream of the *vsi* promoter and SP^vsi^ was sequenced to verify the in-frame fusion of the gene with the *vsi* signal peptide-encoding sequence and to verify the correct coding sequence.

To facilitate the *E. coli* steps of the cloning procedure into pIJ486 [17], the vector was modified and converted into the shuttle-vector pIJ486_Trueblue. This was done by cutting pIJ486 with *Eco*RI and *Bam*HI and ligating the linear plasmid with pTrueBlue (genomics one) that was previously digested with *Eco*RI and *Bam*HI (Additional file 1: Figure S2). Subsequently, high amounts of DNase-free pIJ486_Trueblue DNA were produced in *E. coli*. This DNA was used for the cloning of the target gene. The *E. coli* replication unit was however removed by cleavage concomitant with the insertion of the target genes prior to transformation of *Streptomyces*.

The *vsi* expression/secretion cassette from pBSDK0.6Sma [12] was isolated as a *Bam*HI*/Mfe*I restriction fragment and ligated into pIJ486 following *Bam*HI and *Eco*RI digestion of pIJ486_Trueblue to generate plasmid pIJ486_*vsi*-*celA*. Ligation mixtures were introduced in *S. lividans* by PEG-mediated protoplast transformation [18] and selection for thiostrepton antibiotic resistance. Clones containing *celA* were verified by colony PCR using the cloning primers *celA*-*dsp*-*f* and *celA*-*pst*-*mfe*-*r*.

### Bacterial growth and fermentation

Recombinant *Streptomyces* growth was in the presence of thiostrepton (10 µg/mL) to select for maintenance of plasmid pIJ486_*vsi*-*celA*. Media used in this study as described in [9] were: Phage medium [19] (per liter: 10 g glucose, 5 g tryptone, 5 g yeast extract, 5 g Lab Lemco powder, 0.74 g CaCl_2_·2H_2_O, 0.5 g MgSO_4_·7H_2_O, pH: 7.2), minimal medium (MM) [per liter: 10 g glucose, 3 g (NH_4_)_2_SO_4_, 2.6 g K_2_HPO_4_, 1.8 g NaH_2_PO_4_, 0.6 g MgSO_4_·7H_2_O, 25 mL] minor elements solution (per liter: 40 mg ZnSO_4_·7H_2_O, 40 mg FeSO_4_·7H_2_O, 40 mg CaCl_2_, 40 mg MnCl_2_·4H_2_O), minimal medium with either 5 g/L (MM_C5_) or 15 g/L (MM_C15_) bacto casamino acids, tryptic soy broth (TSB) (per liter: 30 g) [containing 17 g casein peptone (pancreatic)], [5 g NaCl, 3 g soya peptone (papain digest), 2.5 g K_2_HPO_4_, 2.5 g glucose], nutrient broth (NB) without NaCl [per liter: 8 g nutrient broth pH 6.9 (containing 5 g/L peptic digest of animal tissue, 3 g/L beef extract)], double strength nutrient broth (NB_2X_) without NaCl [per liter: 16 g nutrient broth pH 6.9 (containing 10 g/L peptic digest of animal tissue, 6 g/L beef extract)] and Bennet medium (Ben) (per liter: 10 g glucose, 2 g tryptone, 1 g yeast extract, 1 g beef extract). Shake-flask studies were conducted in 2 L Erlenmeyer flasks containing 1 L liquid medium (at 180 rpm; 28 °C) in a New Brunswick 44R temperature-controlled incubator. Fermentation was carried out in an Eppendorf DASGIP Parallel Bioreactor System using 2.3 L vessels containing 1 L medium (at 30 °C; fixed stirring at 500 rpm; pH 6.8 maintained with 4 M KOH and 2 M H_2_SO_4_; air supply of 1 sL/min).

### Protein secretion assays

Western blotting was done using Trans-Blot^®^ SD Semi-Dry Transfer Cell (Bio-Rad). Purified CelA protein served as reference for calibration purposes. Blotted membranes were washed and incubated overnight with the primary antibody. After washing the membrane, the secondary antibody (Jackson Immuno Research) was applied for 1 h. Detection was carried out using the GE Healthcare Amersham ECL reagents and ImageQuant LAS 4000 imager. High resolution images were processed using ImageJ.

Dot-blots were performed using the Bio-Rad Bio-Dot Microfiltration Apparatus according to the manufacturer’s instructions. A nitrocellulose Amersham Protran 0.2 membrane was sandwiched in the manifold and tightened under vacuum followed by washing with TBS buffer and gravitational filtration of diluted supernatants from reactor samples. Purified CelA protein served as reference for calibration purposes. Blotted membranes were treated and detected as previously described in western blotting.

### Protein purification, chromatography and characterization

All chromatography resins and molecular weight markers were from Amersham. Purified proteins were stored at − 20 °C.

For CelA purification from *S. lividans* culture supernatants, cells were grown for 2 days in 500 mL Phage medium and then transferred in 4 L NB for 48 h.

#### Step 1: protein concentration

The *S. lividans* culture supernatants of the 4 L nutrient broth (NB) were concentrated into 200 mL using a rotary evaporator (HeidolphHei-VAP) at 25 °C.

#### Step 2: ammonium sulfate fractionation

Polypeptides in 200 mL of concentrated spent growth medium supernatant were precipitated by gradual slow addition of finely ground (NH_4_)_2_SO_4_ (25% then 55% saturation; 4 °C) and collected by centrifugation (Sorvall RC, F14-6 × 250y rotor, 4 °C, 20 min, 13,000 rpm). Pellets harvested by centrifugation containing CelA as a main polypeptide species and were resuspended in 4 mL of buffer A (50 mM sodium phosphate buffer pH 7.0).

#### Step 3: ion exchange chromatography

The 55% saturation ammonium sulfate fraction from Step 2 containing CelA was washed with 200 mL of buffer A using a Sartorius Vivaspin^®^ 20 centrifugal concentrator (molecular weight cut-off 3000 Da) to remove the salt. The protein pellet was resuspended in 4 mL of buffer A and loaded on a Q-Sepharose column (2 mL) (equilibrated with buffer A). The column was washed with 5 column volumes of buffer A and proteins were eluted with a 0.05–1 M NaCl gradient. The fraction with the highest CelA activity was further treated by heating it at 80 °C for 4 h to remove host contaminants.

### Gel permeation chromatography, circular dichroism and thermal stability

Gel permeation chromatography was as described [20]. CD spectra were recorded on a Jasco J-1500 spectrometer that was equipped with a Peltier-temperature controlled cuvette holder, as described [21, 22]. The thermal denaturation curve of CelA was obtained my monitoring helicity at 222 nm, as a function of temperature (4–82 °C; 0.8 °C/min; slits: 2.5/20). The apparent melting temperature (Tm_app_) was derived by the first derivative of the melting curve, using Prism v4.0 (GraphPad).

### Cellulase functional assay

CelA activity was measured by following the hydrolysis of CMC (carboxy methyl cellulose) [23, 24]. 200 μL substrate (1% w/v CMC in 0.2 M sodium phosphate buffer pH 7.0) was incubated with 0.5 μg of enzyme at 70 °C for 30 min. Subsequently, 300 μL of a DNS solution [per 100 mL: 1 g of 3,5-dinitrosalicylic acid (DNS), 1.6 g NaOH and 30 g K-Na-Tartrate] was added to the reaction mix and the solution was boiled for 5 min. The absorbance of the reaction mix was measured at 546 nm in a microtiter plate reader (Tecan Austria GmbH, Infinite 200) and the results were compared to a standard curve of glucose amounts (0.2–1.4 µmol) and absorbance linearly correlated. One unit of cellulase activity is defined as the amount of enzyme that produces 1 µmol of glucose in 1 min [13]. The catalytic activity was compared to that of a commercial crude preparation of cellulase-containing secretomes from *Aspergillus niger* (0.8 U/mg total crude secretome; measured at 37 °C) (22178 Sigma^®^). Cellulase units of the crude secretome from *A. niger* were calculated by assuming the amount of the unpurified main cellulase (of ~ 27 kDa) to be 1 µg in the total secretome via loading the total secretome on SDS-PAGE (15 µg/lane) and staining by Coomassie blue.

### Miscellaneous

Chemicals were from Sigma. DNA enzymes were from New England Biolabs and oligonucleotides from Eurogentec. Statistical analysis was performed in R language. Differential secretion of CelA was tested using an unpaired t test without assumption of equal variance and p values were adjusted for multiple hypothesis testing error using the Benjamin Hochberg method [25]. Significant difference in secretion was considered when the adjusted p value was < 0.05. Protein aggregation was determined after breaking the cells by sonication, followed by low spin centrifugation to remove unbroken cells (10,000×*g*) and ultracentrifugation (at 100,000×*g*) and detection of proteins by western blotting.

## Results

### Production of recombinant _Rm_CelA in *S. lividans*

**Fig. 1:**
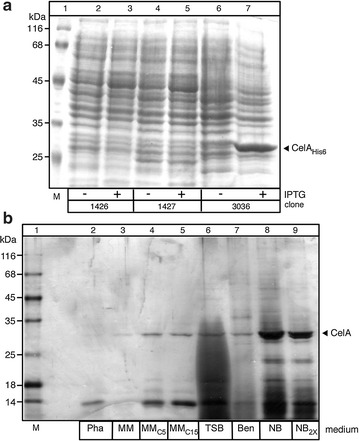
Heterologous synthesis of CelA in *E. coli* and synthesis/secretion in *S. lividans.* **a** CelA_His6_ was synthesized in BL21 (DE3) cells. Expression of CelA_His6_ carried on a pET vector was induced by 0.2 mM IPTG for 3 h at 30 °C. **b** Synthesis in and secretion of CelA from *S. lividans*. Polypeptides (1–8 µg/lane) from culture supernatants (~ 0.1 mL) that is equivalent to 0.2 mg of dry cell mass. The samples from cells grown for 2 days in the indicated media (see “Methods”) were harvested by precipitation with TCA (25%), analyzed by SDS-PAGE and stained with Coomassie blue. Lane 1, molecular weight markers: β-galactosidase (116 kDa), bovine serum albumin (66.2 kDa), ovalbumin (45 kDa), lactate dehydrogenase (35 kDa), restriction endonuclease *Bsp98I* (25 kDa), β-lactoglobulin (18.4 kDa), lysozyme (14.4 kDa). Pha, phage medium; NB, nutrient broth; NB_2X_, nutrient broth double strength; MM, minimal medium; C_5_ and C_15_, casamino acids (5 or 15 g/L); TSB, tryptic soy broth; Ben, Bennet medium

High-level production of CelA was originally achieved by cloning the *celA* gene in *E. coli* in a pET23bAH vector with a C-terminal hexahistidinyl tag [13]. Over-expression of *celA*_*His6*_ in BL21 strains harbouring the T7 polymerase gene was poor in many of the clones tested (e.g. Fig. 1a, lanes 3 and 5). When expressed (lane 7), a percentage of the protein showed a tendency to aggregate (not shown). To explore an alternative production approach and to take advantage of extracellular production of CelA, we used *S. lividans* TK24 with CelA produced as a secreted polypeptide. We successfully used this system previously for the production of tumor necrosis factor α [5] and a 100 kDa xyloglucanase [1]. This approach simplifies downstream protein purification steps and can be optimized at the level of bio-processing. CelA was fused C-terminally to the Vsi signal peptide and placed behind the *vsi* promoter to generate pIJ486-*vsi*-*celA* (Additional file 1: Figures S1, S2).

*Streptomyces lividans* TK24 cells harbouring pIJ486-*vsi*-*celA* were grown in 3 defined and 4 rich growth media using shake-flask cultures or lab-scale 1 L fermenters (Fig. 1b). In all cases, during growth the pH of the culture was controlled and maintained stably at 6.8 during fermentation. Supernatants were harvested at the end of their exponential phase and secreted peptides analyzed by SDS-PAGE and silver staining.

As noted previously [5], significant variation was observed in the overall protein profile of the spent growth media. Cells grown in Phage (Fig. 1b, lane 2) and Bennet (lane 7) media showed little or no secretion of CelA, moderate secretion was seen with the minimal media and TSB (lanes 3–6), while significant levels of secreted CelA were detectable in the spent NB and NB_2X_ media (lanes 8 and 9, respectively). Recombinant CelA migrated with an apparent mass of ~ 26 kDa in SDS-PAGE gels in close agreement with the predicted size of its processed, signal-less mature domain (26 kDa) and represented ~ 50% of extracellular protein in the best producing medium (lane 8). The observed mass is suggestive of correct processing of the polypeptide and removal of the SP^vsi^ as seen commonly with other proteins secreted in this system [1, 5]. The protein was stable in the spent growth medium at 4 °C for several days. In some cases, a minor cleavage was evident that had no effect on enzymatic activity (see below).

### Kinetics and optimal conditions for CelA secretion in *S. lividans*

**Fig. 2:**
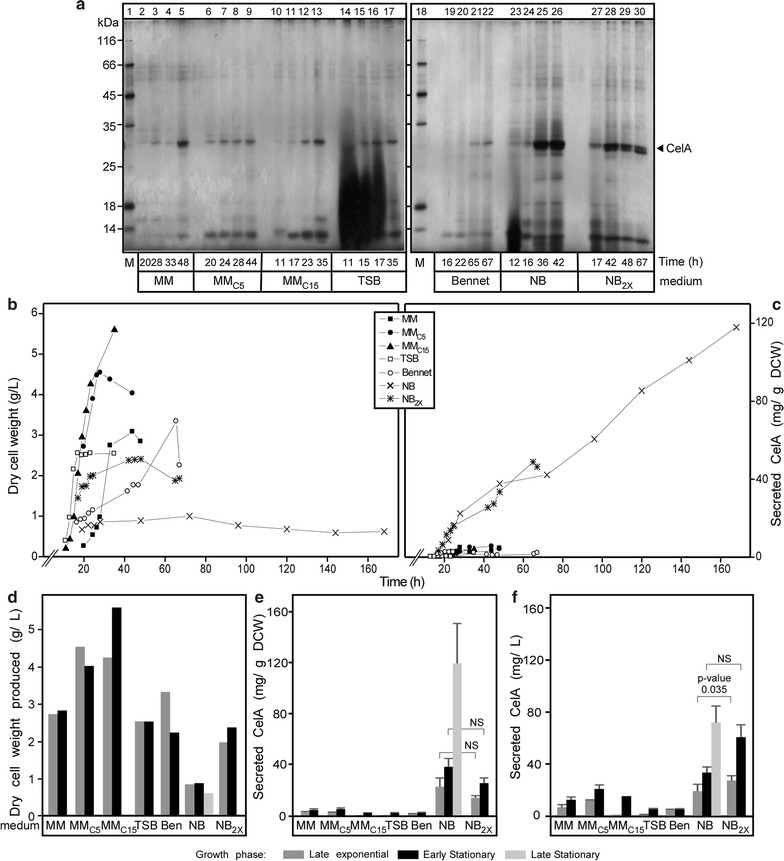
Time course of CelA secretion and the effect of additional carbon source. **a** Polypeptides (0.1–5 µg/lane) from culture supernatants (0.9–16 µL/lane) that is equivalent to (0.08 mg of dry cell mass) from cells grown for the indicated times in the indicated media were analyzed by SDS-PAGE and silver-stained. Lane 1, MW markers as in Fig. 1b. Secreted CelA (filled arrow) is indicated. **b** The growth curves of *S. lividans* TK24 carrying pIJ486 expressing SP^Vsi^-CelA in the indicated media expressed as values of dry cell weight. **c** The dry cell weight in gram per liter produced by *S. lividans TK24* with pIJ486 expressing SP^Vsi^-CelA in the different media (as in Fig. 1b) at the indicated growth phases. Ben, Bennet medium. **d** The amount of CelA secreted (mg) correlated to a gram of dry cell mass by *S. lividans* TK24 with pIJ486 expressing SP^Vsi^-CelA in the different media (as in Fig. 1b) for the indicated time related to its growth curves in the same media. **e**, **f** The amount of CelA secreted (mg) correlated to a gram of dry cell mass (**e**) or 1L of culture (**f**) produced by *S. lividans* TK24 with pIJ486 expressing SP^Vsi^-CelA in the different media at late exponential, early stationary and late stationary phases from **d** difference in CelA secretion was compared using an unpaired t test without assumption of equal variance (see “Methods”; NS, non-significant difference). Error bars represent standard error of the mean (SEM). n = 3

To determine optimal culture conditions and the phase that is productive for secretion, time-course experiments were performed under stably controlled pH (Fig. 2a). In NB, the amount of secreted CelA increased with time and was the highest observed yield of secreted CelA compared to other media (~ 40 mg/L) in 42 h (lanes 25 and 26). Optimal time and yields varied in the other media (Fig. 2a; Table 1), with two consistent observations: (a) biomass accumulation displayed completely different kinetics even when the different rich media were compared (Fig. 2b, d; Table 1) and yielded varying amounts of CelA at the end of the exponential phase (Fig. 2c, e and f). (b) Total endogenous protein secretion and specifically secretion of heterologous CelA, measured using dot-blots or western blots with an α-CelA antiserum, was anti-correlated with increased biomass production (panel d) and this is characteristically seen when CelA secretion from a unit of cell mass is compared (panel e). Contrary to previous observations [5, 26, 27], final CelA secretion yield was not significantly increased by addition of additional components of the NB medium, although the total yields per liter were enhanced (Fig. 2a, lanes 27–30; b, e and f). That is due to increase the amount of biomass produced in NB_2X_ which means the amount of CelA secreted per unit of biomass decreases than NB which produce the less biomass.

**Table 1:** Cellulase A production and yield in different fermentation media over different phases (exponential growth phase versus early stationary phase and late stationary phase)

Medium	End of Exponential phase			Early stationary phase			Late stationary phase		
	CelA concentration (mg/L)	Yield of CelA on biomass (mg/g_DCW_)	Fermentation time (h)	CelA concentration (mg/L)	Yield of CelA per unit of biomass (mg/g_DCW_)	Fermentation time (h)	CelA concentration (mg/L)	Yield of CelA per unit of biomass (mg/g_DCW_)	Fermentation time (h)
MM	6.8	2.5	33	12.9	4.5	48	–	–	–
MMC_5_	12.3	2.7	28	22.0	5.5	44	–	–	–
MMC_15_	1.5	0.4	23.3	15.7	2.8	35	–	–	–
TSB	1.1	0.4	17	5.4	2.1	35	–	–	–
Bennet	4.9	1.5	65	4.9	2.2	67.2	–	–	–
NB	19.2*	22.4**	28	33.5*	37.7**	48	72.1	119.6	168
NB_2X_	27.5*	13.9**	23	60.8*	25.2**	42	–	–	–

The italics numbers indicate the difference in CelA amounts produced in NB and NB_2x_ media

MM, minimal medium; C_5_ and C_15_, casamino acids (5 or 15 g/L); TSB, tryptic soy broth; NB, nutrient broth; NB_2x_, nutrient broth double strength; DCW, dry cell weight. *n* = 3

* The changes in CelA amount in (mg/L). The amount significantly increases with doubling the amount of media

** The changes in CelA amount in (mg/ g DCW). The amount of CelA significantly decreases with doubling the amount of the media

### Effects of CelA secretion on the secretome

**Fig. 3:**
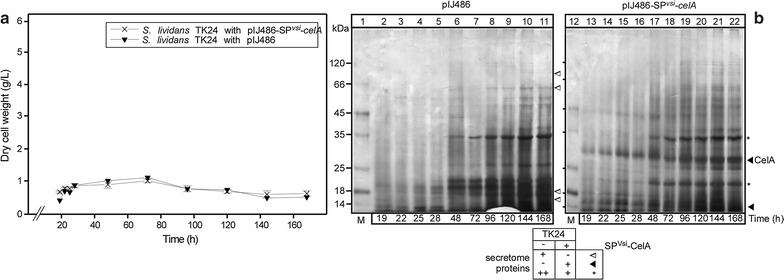
Time course of *S. lividans* TK24 with pIJ486-*vsi*-*celA* and *S. lividans* TK24 with pIJ486 (as a wild type) growth in NB medium. **a** The growth curves of *S. lividans* TK24 carrying empty pIJ486 or pIJ486 expressing SP^Vsi^-CelA in the nutrient broth expressed as values of dry cell weight. **b** Polypeptides (1.2–6 µg/lane) from culture supernatants (~ 7 µL/lane; equivalent to 0.08 mg of dry cell weight) from cells grown for the indicated times in NB were analyzed by SDS-PAGE and silver-stained. Lanes 1 and 12, MW markers as in Fig. 1b. Secreted CelA (filled arrow) is indicated

Time course kinetics experiments in different media (Fig. 2) suggested that the regulation of CelA production and secretion in *S. lividans* TK24 is complex. To gain further insight in the individual roles of the medium and of the heterologously expressed protein we carried out a detailed kinetic comparison between TK24 carrying either an empty vector or one expressing *celA* (Fig. 3). Growth of the two strains was practically identical, thus demonstrating that the NB medium has a fundamental influence on the cell directly, and this leads to acquisition of a stationary phase in ~ 30 h irrespective of whether SP^Vsi^-CelA is produced or not. Moreover, the secretome profile was similar with some notable exceptions (Fig. 3b, marked with symbols). Even in the absence of SP^Vsi^-CelA synthesis, secretome proteins followed obvious growth phase patterns with some coming up early, others late and others not changing. In contrast, SP^Vsi^-CelA was synthesized and secreted very early on in very large amounts making it the most prominent protein of the secretome. Some endogenous secreted proteins showed reduced levels in SP^Vsi^-CelA-producing cells (Fig. 3b, marked with “*”), while others disappeared from the secretome (Fig. 3b, open arrowhead). It is not known whether these proteins compete with SP^Vsi^-CelA for export sites or factors or whether some more complex regulation is in effect.

### Purification of CelA secreted from *S. lividans*

**Fig. 4:**
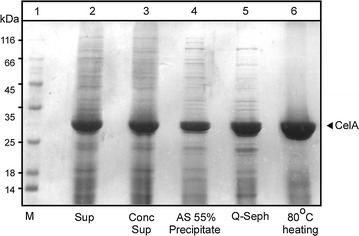
Large-scale purification of *R. marinus* CelA expressed and secreted in *S. lividans* culture supernatants. Protein samples (15 µg/lane) from the various purification steps (see “Methods”) were analyzed as in Fig. 2a and were stained with Coomassie blue. Lane 1: mass markers as in Fig. 1. Lane 2: culture supernatants from *S. lividans* TK24 with pIJ486 expressing SP^Vsi^-CelA grown in NB for 48 h; lane 3: the culture supernatant after concentration with rotary evaporator; lane 4: The 35–55% ammonium sulfate fraction; lane 5: the elution fraction with the highest CelA activity from Q-Sepharose; lane 6: the fraction from lane 5 after heating at 80 °C for 4 h and centrifugal removal of precipitates

We next developed a simple 3-step purification scheme (Fig. 4; see “Methods”) for CelA secreted by *S. lividans* (lane 2). This involved concentration of spent growth media by rotary evaporation (lane 3) and a 25–55% ammonium sulfate fractionation (lane 4), followed by a Q-Sepharose column (lane 5), then heat denaturation at 80 °C (lane 6). More than 80% CelA could be recovered in the supernatant after ultra-centrifugation (lane 5). CelA was purified at more than 98% purity as judged by Coomassie-stained SDS-PAGE gels (lane 6).

### Physical and biochemical characterization of secreted recombinant CelA

**Fig. 5:**
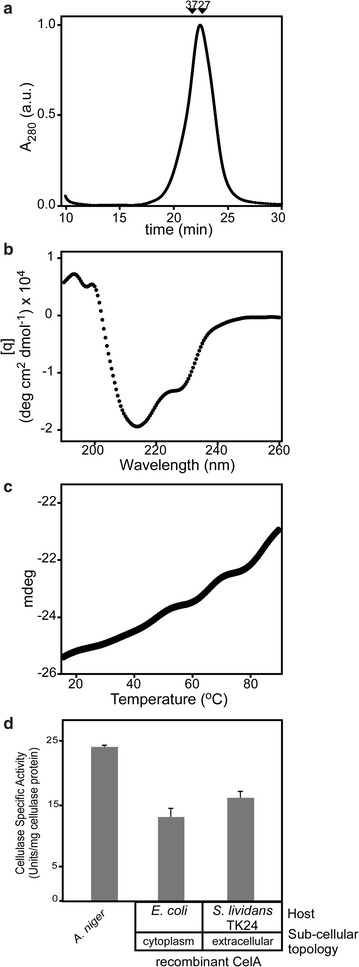
Physical and functional characterization of *S. lividans*-secreted CelA. **a** Size exclusion chromatography of recombinant purified CelA. Arrows indicate migration positions for 35 kDa (CesAB/EspA) and 27 kDa (CesAB) [39]. **b** Circular dichroism spectrometry. 5 µM or protein in buffer (5 mM MOPS pH 7.5, 5 mM NaCl, 1 mM DTT) 20 °C was analyzed using a 190–260 nm wavelength scan. **c** Thermal denaturation curves monitored by circular dichroism. Purified CelA (5 µM) in buffer (5 mM MOPS pH 7.5, 5 mM NaCl, 1 mM DTT) was exposed to gradual temperature rise and changes in ellipticity were monitored at 213 nm, as described [22]. **d** Biochemical activity of *S. lividans*-secreted CelA. Cellulase activity by CelA (20 µg/mL) was determined by hydrolysis of carboxymethyl cellulose (CMC) (see “Methods”). The activity of CelA secreted and purified from *S. lividans* was compared to CelA_His6_ produced in *E. coli* and to a commercial preparation from *A. niger* of 24 U/mg total protein estimated to contain ~ 15 µg of unknown cellulases. *n* = 3; values represent mean ± SD

To evaluate the quality of secreted CelA produced by *S. lividans* we determined whether it was structurally and functionally intact. Analytical size exclusion chromatography revealed that purified secreted CelA represents a monodisperse population without any visible signs of aggregation, with a native apparent molecular weight of ~ 27–30 kDa (Fig. 5a). Recombinant secreted CelA shows a main minimum in circular dichroism spectra at 213 nm, consistent with acquisition of extensive β-stranded secondary structure (Fig. 5b). It is a very stable enzyme with a non-measurable apparent *T*_*m*_ during a temperature gradient of 15–95 °C, carried out by coincident monitoring of ellipticity by circular dichroism (Fig. 5c). This is in agreement with the thermal stability of the native protein’s enzymatic activity [23].

We next examined the catalytic activity of recombinant secreted CelA. The enzyme is highly active in hydrolyzing carboxymethyl cellulose at 70 °C. Despite the presence of secreted wild-type cellulases in TK24 (Additional file 1: Table S1), they displayed no detectable activities at this temperature. The catalytic properties of CelA are in agreement with what was previously determined for the native protein and for CelA_His6_ expressed in *E. coli* [13]. The catalytic activity was compared to that of a crude commercial cellulase-containing *A. niger* secretome, measured at 25 °C as a qualitative control.

We conclude that CelA produced as a secreted polypeptide from *S. lividans* cells is structurally intact and enzymatically fully functional.

## Discussion

We present a pilot scheme for lab-scale production and secretion of functional thermostable cellulase CelA by *S. lividans*. Use of this expression-secretion system was prompted on one hand by failure to establish satisfactory high-level expression of the enzyme using the traditional bacterial host *E. coli* and on the other by a desire to exploit the advantages of protein secretion from *S. lividans* in downstream processing. Use of *S. lividans* is an excellent alternative host strategy for protein production and production of CelA strengthens its specific application in protein secretion biotechnology.

CelA can be produced at high levels from *S. lividans* under specific fermentation regimes where the secreted form represents > 50% of the total protein present in the top performing spent growth media (Figs. 1b, 2). *Rhodothermus marinus* belongs to the *Cytophaga*–*Flavobacterium*–*Bacteroides* phylogenetic group and despite the evolutionary distance between the two organisms (*S. lividans* belongs to the *Actinomycetales*), high level expression of the *R. marinus celA* gene in *S. lividans* was not impeded. *S. lividans* also encodes and synthesizes multiple cellulase enzymes (Additional file 1: Table S1), like the highly homologous CelB2 [14], but these do not seem to be generally active under our growth and assay conditions and are not detected in spent growth media by mass spectrometry (Tsolis and Hamed, unpublished). Our previous attempts to secrete from *S. lividans* heterologous proteins that were carrying their own native signal peptides were unsuccessful [1] and so we systematically resort to using a native streptomycetal signal peptide for secretion. This reconfirms the importance of appropriate signal peptide mature domain combinations for optimal heterologous secretion as they are essential in regulating the cooperative binding of the preprotein to SecA as a bivalent ligand [7, 8, 22, 28].

A secretion yield of ~ 70 mg/L seen with small-scale cultures or lab-scale fermentors is among the best obtained to date from this host organism and may be further improved with bioprocess optimization. The best yields obtained to date include murine tumor necrosis factor alpha (80–120 mg/L) [5], xyloglucanase (100–150 mg/L) [1] and APA protein (alanine and proline-rich secreted protein) (80 mg/L) [29]. Significant amounts of CelA are observed after 7 days of culture and accumulated protein was observed after prolonged incubations suggesting that secreted CelA is generally proteolytically stable and remains unaffected by *S. lividans* secreted proteases. This was commonly observed for many heterologous proteins secreted by *Streptomyces* [2, 5, 30, 31] but not for others [32]. Addition of optimal carbohydrate amounts can lead to maximal product yields at reduced culture times [5]. The fermentation pattern we saw with CelA secretion was more complex (Fig. 2). Various carbon sources in the media caused reduction of CelA synthesis and/or secretion (Fig. 2). The expression/secretion of some secreted polypeptides was also affected, while that of others was not (Fig. 2a). Notably, optimal secretion was correlated with reduced media-specific growth (e.g. NB medium) and not seen with all other rich media. This suggests that certain components in one rich medium drive biomass production, while in other media, although rich, a medium component is in low abundance and presumably becomes quickly depleted. This prevents further biomass production and switches the cells to a stationary-phase state in which protein secretion is maximized. The medium components responsible, the genetic regulatory mechanisms that are responsible for switching and how protein secretion is linked to such stationary phase regulation remain unknown. We characteristically observe that certain polypeptides will begin production/secretion late into stationary phase (Fig. 3b), indicating that different levels of stationary phase control elements may exist. These findings may provide a first means to identifying the underlying metabolic connection to the secretome by using poly-omics approaches, currently under development. This and other studies [27, 33, 34] indicate that growth conditions may exert a degree of regulation of the secretory protein genes and/or the secretion pathway genes of *Streptomyces*. Such regulation is not known to exist in the *E. coli* system, where many Sec component genes are found in ribosomal operons.

It is evident that to further develop *Streptomyces* secretion biotechnology it is important to understand how different fermentation regimes affect protein secretion of native and heterologous proteins and this asks for a combination of transcriptomics, proteomics and fluxomics approaches. For this, the determination of the *S. lividans* TK24 genome [35], its comprehensive annotation and transcriptome determination (Busche et al. in preparation), the determination of the secreted proteome and its dynamics (Tsolis et al. in preparation), the determination of metabolomics tools [36] and the optimization of micro-scale culture arrays for robust biological reproducibility regimes [37] are important first steps. TK24 expressing CelA provides a suitable model to test these parameters.

The varying profiles of endogenous *Streptomyces*-secreted proteins under different growth conditions (Fig. 2a), makes the establishment of strict culture conditions very important for reproducibility of subsequent purification schemes. The production of highly-expressed endogenous *S. lividans* proteins such as the subtilisin inhibitor of ~ 14 kDa (Fig. 2a) could compete with secretion of the heterologous protein of interest and should be controlled either at the level of their synthesis or through genetic means (e.g. gene deletion).

CelA secreted by *S. lividans* is a stable polypeptide and is highly active in biochemical assays (Fig. 5d). Cellulose is one of the most abundant polysaccharides in nature and cellulases are important industrial enzymes with potential application in the feed industry, in the paper industry and for improving conversions in biofuel production [38]. The successful expression of this thermostable cellulase as a secretory protein from *S. lividans* described here is compatible with many of the established processes of industrial enzyme biotechnology and opens new areas for industrial application of cellulases.

The good secretion yield of CelA suggests that the *S. lividans* cell factory may be well suited for large-scale production of other thermostable polypeptides of industrial importance.

## Additional file

**Additional file 1: Figure S1.** Amino acid sequence of cellulase A (CelA). **Figure S2.** pIJ486_*Vsi-celA* plasmid. **Table S1.** Annotated secreted cellulase-related hydrolases in the *S. lividans* TK24 proteome.
