# Supplementary material for: Large-scale production of a thermostable Rhodothermus marinus cellulase by heterologous secretion from Streptomyces lividans
Source: Microb Cell Fact. 2017 Dec 23;16:232. doi: 10.1186/s12934-017-0847-x (PMC5741968; doi:10.1186/s12934-017-0847-x)
Supplement: Supplementary file 1 — Additional file 1: Figure S1. Amino acid sequence of cellulase A (CelA). Figure S2. pIJ486_Vsi-celA plasmid. Table S1. Annotated secreted cellulase-related hydrolases in the S. lividans TK24 proteome. [file 12934_2017_847_MOESM1_ESM.pdf]

Additional file 1:

Large-scale production of a thermostable *Rhodothermus marinus* cellulase by heterologous secretion from *Streptomyces lividans*

Hamed, Mohamed Belal[1,4]; Karamanou, Spyridoula[1]; Ólafsdottir, Solveig[2]; Basílio, Joan Sofia Martins [3]; Simoens, Kenneth[3]; Tsolis, C. Kostantinos[1]; Van Mellaert, Lieve[1]; Guðmundsdóttir, Eik Elísabet[2]; Hreggvidsson, Gudmundur Oli[2]; Anné, Jozef[1]; Bernaerts, Kristel[3]; Fridjonsson, H. Olafur[2] and Economou, Anastassios[1,5]

[1]KU Leuven, Rega Institute, Dpt of Microbiology and Immunology, Herestraat 49, B-3000 Leuven, Belgium
[2]Matís, Vínlandsleid 12, 113 Reykjavík, Iceland
[3]KU Leuven, Bio- & chemical systems Technology, Reactor Engineering and Safety Section, Department of Chemical Engineering, Celestijnenlaan 200F, 3001 Leuven, Belgium.
[4] Department of Molecular Biology, National Research Centre, Dokki, Giza, Egypt

[5] For correspondence,
E-mail: tassos.economou@kuleuven.be

Table of contents

Additional Figures

Figure S1 Amino acid sequence of cellulase A (CelA)

Figure S2 pIJ486_*Vsi-celA* plasmid.

Additional Tables

Table S1 Annotated secreted cellulase-related hydrolases in the *S. lividans* TK24 proteome.

References

```
                                        -3-1
  1 MRRTLKAVGA AAAAATCVLA ATAGTAQAEA KEPEPEPEPT VELCGRWDAR DVAGGRYRVI
 61 NNVWGAETAQ CIEVGLETGN FTITRADHDN GNNVAAYPAI YFGCHWGACT SNSGLPRRVQ
121 ELSDVRTSWT LTPITTGRWN AAYDIWFSPV TNSGNGYSGG AELMIWLNWN GGVMPGGSRV
181 ATVELAGATW EVWYADWDWN YIAYRRTTPT TSVSELDLKA FIDDAVARGY IRPEWYLHAV
241 ETGFELWEGG AGLRSADFSV TVQ
```

Fig. S1 Amino acid sequence of cellulase A from *Rodothermus marinus*

Amino acid sequence of cellulase A (CelA) from *Rodothermus marinus* fused to signal peptide of Vsi (SP^{Vsi}), the subtilisin inhibitor of *Streptomyces venezuelae* (Uniprot: SSI_STRVL); highlighted in red).The cleavage site (-3 and -1) of the Vsi signal peptide predicted by SignalP4.0 is included at Alanine 26 and Alanine 28 of the sequence. Two additional amino acids of the mature Vsi domain (Glu-Ala, underlined) were maintained in the SP^{Vsi}-CelA fusion protein. The CelA sequence starts from lysine at position 30 (bold K)(residue 29 in the original CelA from *Rhodothermus marinus* sequence (Uniprot: O33897_RHOMR).

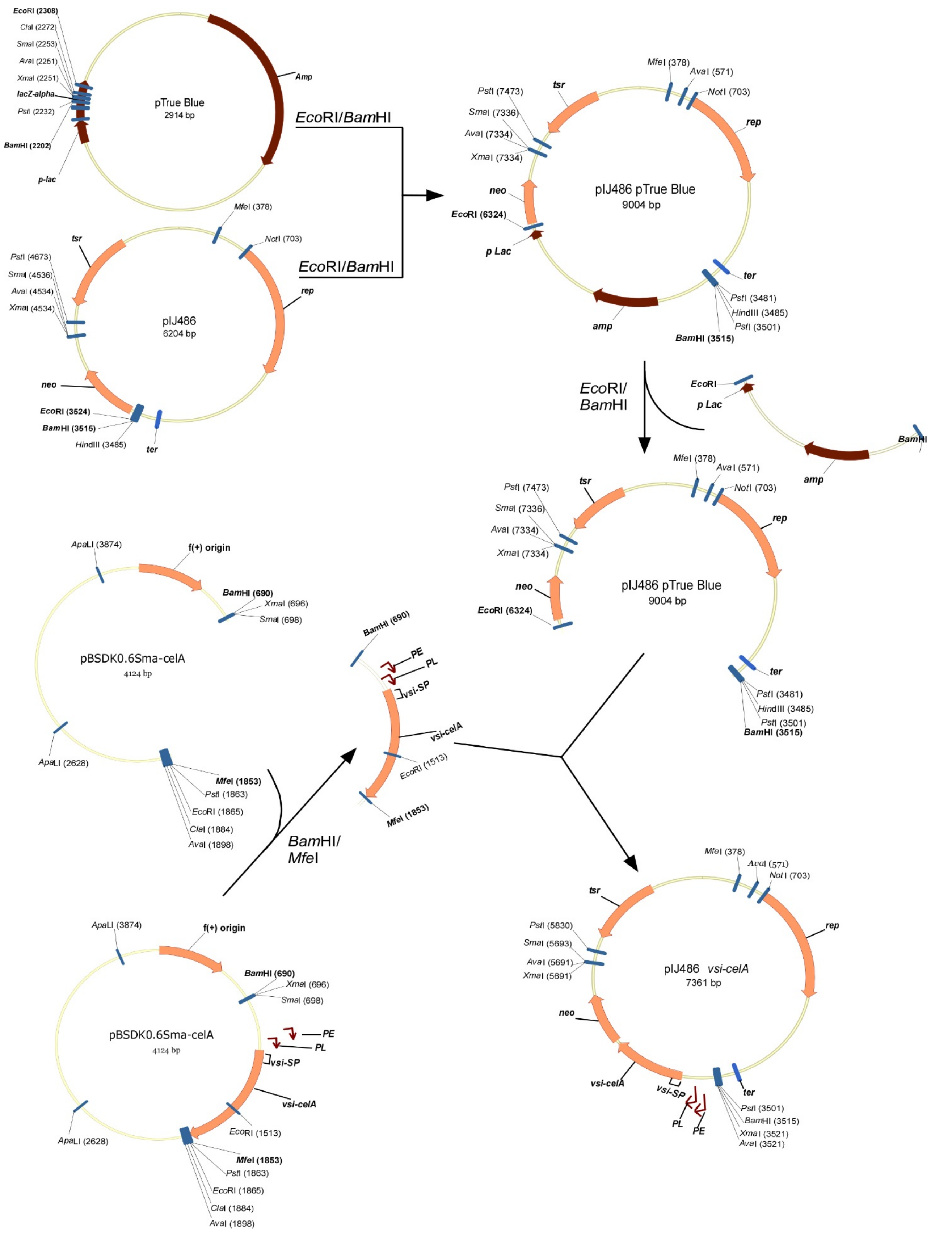

Fig. S2. Scheme of cloning of *vsi-celA in pIJ486*

Vector pIJ486 [1] was modified and converted into the *Streptomyces-E.coli* shuttle-vector pIJ486_pTrueBlue by digesting with *Eco*RI and *Bam*HI and ligating the linear plasmid with *Eco*RI and *Bam*HI-digested pTrueBlue. This was to facilitate recombinant DNA handling steps in *E. coli*. The *E. coli* replication unit was removed from DNAse-free pIJ486_pTrueBlue DNA produced in *E. coli* before transformation to *Streptomyces* with the same restriction enzymes used for cloning. The *vsi* expression/secretion cassette was derived from pBSDK0.6Sma [2] and was isolated as a *BamHI/MfeI* restriction fragment and ligated into *BamHI* and *EcoRI*-digested pIJ486_pTrueBlue to generate plasmid pIJ486*vsi-celA*.

Table S1 Annotated secreted cellulases-related hydrolases in *S. lividans TK24*.

Proteome annotation of *S. lividans TK24*, revealed 14 secreted cellulases (SToPSdb, Tsolis et al., in preparation; www.stepdb.eu/slividans/slividanst24list.php). Five of them have been experimentally detected using mass spectrometry, in various proteomics studies performed in our lab. N/A, not available

Gene ID	Gene name	Uniprot Accession	Protein name (SToPSdb)	Experimental detection by mass spectrometry/reference
SLIV_23465		D6EWL9	Secreted lipoprotein (TAT) - Glucanase	YES/[3]; (Tsolis et al., in preparation)
SLIV_01130	celA	D6ESW5	Secreted protein (Sec) - Endoglucanase CelA	YES/(Tsolis et al., in preparation)
SLIV_06970	manA	A0A076M3U4	Secreted protein (Sec) - Mannan endo-1,4-beta-mannosidase	YES/(Tsolis et al., in preparation))
SLIV_05185		D6EGJ2	Secreted protein (TAT) – Glucosidase	YES/(Tsolis et al., in preparation)
SLIV_01430		D6EU74	Secreted protein (Sec) - Glucan endo-1,3-beta-glucosidase	YES/(Tsolis et al., in preparation)
SLIV_05695	cbhB	D6EGU5	Secreted protein (TAT) - Exoglucanase B	N/A
SLIV_34105	cel1	D6EYB4	Secreted protein (TAT) - Glycoside hydrolase family 9 - Endoglucanase	N/A
SLIV_31860	celB	D6ESG9	Secreted protein (Sec) - Cellulase B	N/A
SLIV_01515	glcI	D6EU91	Secreted protein (TAT) - Glucan endo-1,3-beta-glucosidase	N/A
SLIV_05700	xeg74	A0A076LTM1	Secreted protein (Sec) - Xyloglucanase	N/A
SLIV_35170		D6EDG7	Secreted protein (Sec) - Endoglucanase	N/A
SLIV_05690		A0A076LZR2	Secreted protein (Sec) - Glucanase	N/A
SLIV_30510		A0A076MHD6	Secreted protein (Sec) - Glucanase	N/A
SLIV_01395		A0A076LX29	Secreted Lipoprotein (Sec) - Glycoside hydrolase family 9	N/A

References:

1. Ward JM, Janssen GR, Kieser T, Bibb MJ, Buttner MJ: **Construction and characterisation of a series of multi-copy promoter-probe plasmid vectors for *Streptomyces* using the aminoglycoside phosphotransferase gene from Tn5 as indicator.** *Mol Gen Genet* 1986, **203:**468-478.
2. Van Mellaert L, Lammertyn E, Schacht S, Proost P, Van Damme J, Wroblowski B, Anné J, Scarcez T, Sablon E, Raeymaeckers J, Van Broekhoven A: **Molecular characterization of a novel subtilisin inhibitor protein produced by *Streptomyces venezuelae* CBS762.70.** *DNA Seq* 1998, **9:**19-30.
3. Koepff J, Keller M, Tsolis CK, Busche T, Rückert C, Hamed MB, Anne J, Kalinowski J, Wiechert W, Economou A, Oldiges M: **Fast and reliable strain characterization of Streptomyces lividans through micro-scale cultivation.** *Biotechnol Bioeng* 2016**:**(In press).
